# Suicide and other causes of death among working-age and older adults in the year after discharge from in-patient mental healthcare in England: matched cohort study

**DOI:** 10.1192/bjp.2021.176

**Published:** 2021-12-14

**Authors:** Rebecca Musgrove, Matthew J. Carr, Nav Kapur, Carolyn A. Chew-Graham, Faraz Mughal, Darren M. Ashcroft, Roger T. Webb

**Affiliations:** National Institute for Health Research Greater Manchester Patient Safety Translational Research Centre, University of Manchester, UK; Centre for Mental Health and Safety, Division of Psychology and Mental Health, Faculty of Biology, Medicine and Health, University of Manchester, UK; Manchester Academic Health Science Centre, UK; National Institute for Health Research Greater Manchester Patient Safety Translational Research Centre, University of Manchester, UK; Centre for Pharmacoepidemiology and Drug Safety, Division of Pharmacy and Optometry, Faculty of Biology, Medicine and Health, University of Manchester, UK; Manchester Academic Health Science Centre, UK; National Institute for Health Research Greater Manchester Patient Safety Translational Research Centre, University of Manchester, UK; Centre for Mental Health and Safety, Division of Psychology and Mental Health, Faculty of Biology, Medicine and Health, University of Manchester, UK; Manchester Academic Health Science Centre, UK; Greater Manchester Mental Health NHS Foundation Trust, UK; School of Medicine, Keele University, UK; School of Medicine, Keele University, UK; NIHR Greater Manchester Patient Safety Translational Research Centre, UK; Unit of Academic Primary Care, University of Warwick, UK; National Institute for Health Research Greater Manchester Patient Safety Translational Research Centre, University of Manchester, UK; Centre for Pharmacoepidemiology and Drug Safety, Division of Pharmacy and Optometry, Faculty of Biology, Medicine and Health, University of Manchester, UK; Manchester Academic Health Science Centre, UK; National Institute for Health Research Greater Manchester Patient Safety Translational Research Centre, University of Manchester, UK; Centre for Mental Health and Safety, Division of Psychology and Mental Health, Faculty of Biology, Medicine and Health, University of Manchester, UK; Manchester Academic Health Science Centre, UK

**Keywords:** In-patient treatment, epidemiology, discharge, suicide, mortality

## Abstract

**Background:**

Evidence for risk of dying by suicide and other causes following discharge from in-patient psychiatric care throughout adulthood is sparse.

**Aims:**

To estimate risks of all-cause mortality, natural and external-cause deaths, suicide and accidental, alcohol-specific and drug-related deaths in working-age and older adults within a year post-discharge.

**Method:**

Using interlinked general practice, hospital, and mortality records in the Clinical Practice Research Datalink we delineated a cohort of discharged adults in England, 2001–2018. Each patient was matched to up to 20 general population comparator patients. Cumulative incidence (absolute risks) and hazard ratios (relative risks) were estimated separately for ages 18–64 and ≥65 years with additional stratification by gender and practice-level deprivation.

**Results:**

The 1-year cumulative incidence of dying post-discharge was 2.1% among working-age adults (95% CI 2.0–2.3) and 14.1% (95% CI 13.6–14.5) among older adults. Suicide risk was particularly elevated in the first 3 months, with hazard ratios of 191.1 (95% CI 125.0–292.0) among working-age adults and 125.4 (95% CI 52.6–298.9) in older adults. Older patients were vulnerable to dying by natural causes within 3 months post-discharge. Risk of dying by external causes was greater among discharged working-age adults in the least deprived areas. Relative risk of suicide in discharged working-age women relative to their general population peers was double the equivalent male risk elevation.

**Conclusions:**

Recently discharged adults at any age are at increased risk of dying from external and natural causes, indicating the importance of close monitoring and provision of optimal support to all such patients, particularly during the first 3 months post-discharge.

## Background

For people who are admitted to an in-patient mental health ward, the transition back to living in the community can be challenging. Patients may still be extremely unwell and could be returning to difficult life circumstances.^1^ The period shortly after discharge has been identified as a time of greatly elevated suicide risk.^2,3^ Studies have also identified an increased risk of dying by natural causes in people with experience of in-patient psychiatric admission relative to those without such a history.^4^ Although heightened suicide risk after discharge is well established^2,3^ studies often lack a comparison with the general population. No recent longitudinal studies in England have estimated the absolute or relative risk of suicide within the first year of discharge.^2^ Furthermore, published meta-analyses have considered only absolute risk and have not identified significant differences in risk between older adults and working-age groups.^2,5^ Studies investigating other causes of death do not usually focus on the immediate discharge period.^4^ Few studies have examined the relative risk for a range of causes of death occurring within the first year of discharge across both working-age and older adult populations, precluding direct comparison of these risks.^6^

### This analysis

We aimed to address these gaps in the evidence base by estimating, separately for working-age and older adults:

(a) the absolute risks of all-cause mortality, any natural and any external causes of death, suicide and accidental death, and alcohol-specific and drug-related death in the first year after discharge from in-patient mental healthcare;
(b) the relative risk of these outcomes versus people in the general population at 3 months and 1 year after discharge; and
(c) how these risks vary by gender and relative level of neighbourhood deprivation.

We expected the working-age discharged cohort to have much higher risks for all adverse outcomes examined compared with the general population, with especially elevated risk for suicide during the first post-discharge weeks. The patterns of absolute and relative risk among discharged older adults were less predictable in our view. To the best of our knowledge, for the first time in England, routinely collected, interlinked primary and secondary care electronic health records of patients were used to delineate the study cohort, with additional linkage to mortality records. This enabled us to directly compare risks for cause-specific mortality versus people without a recent history of in-patient psychiatric admission. Comparison of absolute and relative risks between working-age and older adults can inform post-discharge and community-based care to ensure that all discharged patients receive effective and tailored support.

## Method

### Data source

The Clinical Practice Research Datalink (CPRD) comprises two data-sets, GOLD and Aurum, consisting of detailed patient records from general practices using Vision® and EMIS Web® electronic record systems, respectively. In the UK over 98% of the population are registered with a National Health Service (NHS) general practice; approximately 20% of this population are included in the CPRD. Patients included in the CPRD data-sets are broadly representative of the UK population’s sociodemographic profile, and are considered to be of high quality. Our study used the combined June 2020 extracts of the data-sets with a ‘bridging’ file excluding any duplicate practices that had migrated from Vision to EMIS Web. The CPRD links GP-registered patients to the Hospital Episode Statistics Admitted Patient Care (HES APC) data-set, which contains information on admissions to English NHS hospitals, to mortality records compiled by the Office for National Statistics (ONS), and to Index of Multiple Deprivation (IMD) quintiles – 2015 English index. The IMD is a composite measure of relative deprivation encompassing health, income, employment, education, crime, barriers to housing and services, and living environment and is measured according to patients’ and practices’ postcodes at the lower layer super output area, each containing 1000–3000 residents. Further information and key references regarding data-sets can be found in the Supplementary Appendix 1 (available at https://doi.org/10.1192/bjp.2021.176).

### Ethical approval and consent

The authors assert that all procedures contributing to this work comply with the ethical standards of the relevant national and institutional committees on human experimentation and with the Helsinki Declaration of 1975, as revised in 2008. All procedures involving human patients were approved by the NHS Health Research Authority’s East Midlands – Derby Research Ethics Committee (reference number 05/MRE04/87), as part of a wider CPRD application to support research using anonymised patient data. The CPRD’s Independent Scientific Advisory Committee approved the protocol in April 2020 (ref. 20_038R). Individual patient consent was not required as data are anonymised and routinely collected, and all patients may opt-out of sharing their data for research.

### Study population and design

People living in England who were discharged from an in-patient psychiatric unit between 1 January 2001 and 31 May 2018 were identified from the HES APC. The first discharge date during the study’s observation period represented the index date for follow-up initiation. This period was chosen to maximise power to estimate suicide risk. Full data linkage is only available from 1998. We chose to start the study in 2001 to provide a sufficient look-back window (3 years) to limit our cohort to those who were being admitted to hospital for the first time, or who had not been recently admitted to hospital. The end of the period was set 1 year earlier than the latest data available to allow for 1 year follow-up. Patients were included if they were at least 18 years old and registered at a CPRD practice at the index date and for 6 months (183 days) before that date. A matched cohort study design was implemented. The comparison cohort consisted of up to 20 people per discharged patient, identified via primary care records, matched to discharged cohort members by registered general practice, exact year of birth and gender. They were excluded if they had received in-patient psychiatric treatment within 3 years before the matched discharged patient’s index date. Only those patient records that were deemed acceptable for research purposes by the CPRD (including valid gender and year of birth) and with linkage eligibility were included. Supplementary Table 1 shows details of the primary cohort delineation and exclusions. The cohort was split into two broad age groups, those of working-age (18–64 years) and older adults (65 years and over). All cohort members were followed up from the index date for up to a year until occurrence of the examined outcome, death by another cause, transfer out of practice or observation period end date (31 May 2019).

### Classification of outcomes and covariates

Cause of death was ascertained via linked ONS mortality records. Suicides, including deaths by external cause of undetermined intent, as is the convention in the UK,^7^ were classified as ICD-10 X60–X84, Y10–Y34 (excluding Y33.9), Y87.0, Y87.2, and accidental death as V01–X59, Y85–Y86. Alcohol-specific and drug-related deaths were classified according to standard ONS groupings (see Supplementary Appendix 1). External causes, a broad category including all deaths by suicide, accident, and interpersonal violence, comprised all Chapter XX codes (V01–Y98) and natural deaths were any other codes. Less than 0.1% of all patient records had a missing patient postcode-derived IMD quintile, in which case the practice’s IMD quintile was applied instead. Coding ranges in ICD-10 for the index in-patient episode primary diagnostic categories were reviewed by clinicians (N.K., C.A.C.-G., F.M.). Non-fatal self-harm was identified in primary care records using a coding range described in a previous CPRD-based study^8^ and in secondary care using hospital admissions for intentional self-harm (codes X60–X84). Length of index hospital admission and type of discharge were identified from the HES records. Existing comorbidities at baseline were classified using code lists based on the Charlson Comorbidity Index (CCI).9 Dementia at baseline was identified in primary care using the relevant CCI codes and from HES in-patient episodes using ICD-10 codes (See Supplementary Appendix 1).

### Data analysis

All analyses were performed using Stata version 16 software.^10^ Cumulative incidence was calculated for each outcome at 1-year post-discharge by age group (18–64, 65 years and older) and gender. Estimates took into account competing risks using Stata commands developed by a statistician (M.J.C.) based on the recommendations made by Gooley et al.^11^ Survival analysis was performed using Cox regression^12^ for each examined outcome, by age group separately, with stratification on the matched sets. Unadjusted hazard ratios (HRs) and HRs adjusted for patient postcode IMD quintiles, were estimated. The matched design precluded confounding by age and gender, and also socioeconomic factors to some degree via matching on area-level deprivation according to practice localities. Cox regression assumes proportional hazards^12^ (i.e. observed relationships remain constant throughout the follow-up period). Previous studies have identified much higher rates of suicide immediately after discharge.^2,3,6^ Where such patterns were identified via a Schoenfeld residuals test and by plotting the scaled residuals, HRs were presented separately according to varying follow-up periods. These analyses were repeated, fitting an interaction term for gender. Final Cox regression models for external and natural causes, and suicide, were fitted limited to the discharged cohort with practice-level IMD quintile as the primary exposure, adjusted for gender and age. Various models were assessed with age treated as linear and non-linear, and by 10-year and broader age bands (18–39, 40–64, 65–74 and ≥75) using Akaike’s and Schwarz’s Bayesian information criteria (AIC and BIC) to assess goodness-of-fit. These options were assessed as the risks of dying from external versus natural causes vary considerably by age.

Sensitivity analyses were run excluding (a) time in subsequent psychiatric admission, (b) older adults with a primary diagnosis of dementia, (c) the final year of discharges, and (d) individuals with no documented overnight stay. Details can be found in the Supplementary Appendix 2.

This study is reported in line with RECORD guidance, extended from the STROBE statement for studies using observational routinely collected data (Supplementary Table 2).

The first author (R.M.) met with an advisory group of mental health service patients and carers who advised on initial and revised plans and gave feedback on the findings and possible interpretations. The advisory group was supported by the NIHR Greater Manchester Patient Safety Translational Research Centre.

## Results

### Descriptive information

The primary cohort consisted of 67 559 discharged patients aged 18–64 years and 33 302 aged 65–105 with 1 899 921 matched comparators. Table 1 indicates the demographic and hospital-related characteristics of the discharged individuals. Their median age was 39 years (interquartile range (IQR) = 19, 75th percentile: 49; 25th percentile: 30) in the working-age cohort and 79 years (IQR = 12, 75th percentile: 84; 25th percentile: 72) in the older cohort, with 45% of working adults and 61% of older adults being women. Discharged patients in both age categories were disproportionately registered with a practice in more deprived areas.

Clinically, the most common primary diagnostic groups in the working-age population were people with mood disorders (18.3%), substance use disorders (18.1%) and schizophrenia and related disorders (17.7%). In the older population, dementia was the most common primary diagnosis (34.8%), followed by mood disorders (20.7%). Around 1 in 7 (13.6%) of all those discharged had an unspecified diagnosis code. In older adults 49.0% of the discharged cohort had a diagnosis of dementia in primary or secondary records compared with 3.8% in the comparison group. A higher percentage (22%) of patients in the discharged cohort (compared with 7% of the comparison group) were censored because of transferring to a new general practice during the 1-year follow-up period.

### Absolute risk in discharged working-age and older adults

A total of 1301 discharged patients aged 18–64 died within a year of discharge, a cumulative incidence of 2.1% (95% CI 2.0–2.3), compared with 0.2% (95% CI 0.2–0.2) in the matched cohort, with over 50% dying of external causes (Table 2). Suicide had the highest absolute risk, accounting for 40% of all deaths in this age category. There were 3473 deaths by any cause among older discharged adults, giving a 14.1% absolute risk in the first year (95% CI 13.6–14.5) compared with 4.8% (95% CI 4.7–4.9) in the matched comparison cohort. Most deaths (94%) in the older discharged group were from natural causes, the most common natural causes being diseases of the circulatory system (34.6%) and dementia (20.1%). The absolute risk of suicide in discharged older adults was less than half that of the working-age group. Only death by natural causes and accidents had a higher cumulative incidence in the older-age discharged cohort (Table 2).

In sensitivity analyses excluding older adults with dementia, the absolute risk of death reduced to 11.1% (95% CI 10.6–11.5), although the median age also dropped from 79 to 76 (Supplementary Table 3).

### Relative risk by time elapsed since discharge

**Fig. 1:**
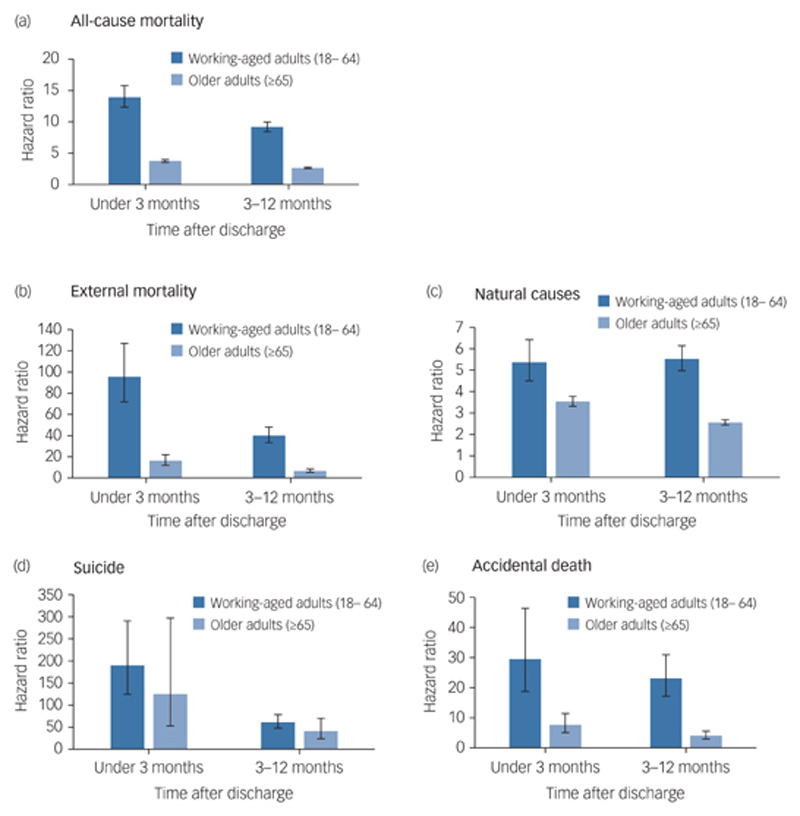
Relative risks for specific causes of death among working-age and older adult patients at under 3 months and 3 months to 1 year after discharge. (a) All-cause mortality; (b) external mortality; (c) natural causes; (d) suicide; (e) accidental death. All analysis adjusted for Index of Multiple Deprivation at patient level. Error bars show 95% confidence intervals.

HRs were higher in the discharged cohorts relative to their general population comparators for all causes examined at 3 months and at 1-year post-discharge, in both age categories (Fig. 1). The elevated risk of death in older discharged adults compared with their matched comparators was lower than for working-age adults, except for suicide, where there was no significant difference in relative risk (Fig. 1). Risks of drug-related and alcohol-specific deaths in older adults were not calculated because of the small numbers, but in working-aged adults the HR in the first three months for drug-related deaths was 84.1 (95% CI 49.7–142.4) and for alcohol deaths, 19.9 (95% CI 12.0–32.8).

All-cause mortality, external causes of death, and suicide showed elevated HRs in the first 3 months after discharge compared with the rest of the post-discharge follow-up year in both age categories (Fig. 1). People aged 18–64 were 191.1 times more likely to die by suicide than their matched comparators (95% CI 125.0–292.0) during the first 3 months, with the equivalent value among discharged older adults being 125.4 (52.6–298.9). In older adults, the HR for natural causes also showed a marked elevation in the 3 months after discharge (HR 3.5, 95% CI 3.3–3.8) compared with 3–12 months (HR 2.6, 95% CI 2.4–2.7), a pattern not seen in the working-age population. This marked elevation in risk of dying by natural causes in the first 3 months, although marginally lower, remained after exclusion of individuals with a primary diagnosis of dementia (see Supplementary Fig. 1).

### Gender-specific risk

Absolute risk of death at 1 year was higher in men than in women in both age groups and for each cause of death category examined (Table 2). However, relative to women of the same age in the general population, across the 1-year follow-up period, discharged working-age women had a greater elevation in risk of death from external causes (*P* = 0.001), accidental death (*P* = 0.044) and suicide (*P* = 0.003) than among discharged men (versus their general population peers; Supplementary Table 4). The larger risk elevation among discharged women was greatest for suicide, with the average first-year hazard of suicide in discharged women being 163.9 times that of women of the same age in the general population (95% CI 100.3–267.7), more than double the equivalent male relative risk value (Supplementary Fig. 2). This pattern was not identified in older adults; the average relative risks of suicide compared with the general population were similar in men 67.7 (95% CI 37.2–123.1) and women 62.9 (95% CI 29.8–132.7). The gender-specific HRs are presented as an average for the year as the proportional hazards assumption did not hold, but data were too sparse to make meaningful comparisons over the first 3 months post-discharge.

### Risk by practice-level deprivation quintile

Finally, although discharged people were disproportionately registered at practices located in deprived areas, working-age adults in the three most deprived quintiles had a lower risk of dying by external causes than those in the least deprived quintile (Table 3).

This was particularly evident with suicide (Supplementary Fig. 3), with discharged working-age patients registered in the most deprived quintile having almost half the risk versus the least deprived (HR 0.57, 95% CI 0.43–0.74). There was no evidence that the relative risk of death by natural causes varied by practice-level deprivation quintile for adults discharged at working age. Conversely, among discharged older adults, the relative risk of natural mortality was greater in the more deprived quintiles (Supplementary Fig. 4).

## Discussion

### Summary of findings

In our matched cohort study, we examined the absolute and relative risks of a range of causes of death in working-age and older adults recently discharged from in-patient mental healthcare in England. Just over 2.1% of working-age (0.2% in the general population) and 14.1% of older adults (4.8% in the general population) died within a year of discharge. Compared with the general population, the risk of death was greater for recently discharged people for each cause of death studied, including suicide, deaths related to drugs and alcohol, as well as accidents and deaths by natural causes. This risk elevation was generally more pronounced in the working-age group than for older adults, although there was no significant difference identified in relative risk of death by suicide. As expected, the relative risk of death by external causes was greatest in the first 3 months post-discharge, most markedly for suicide (191 times higher than the matched working-age comparators). In older adults, the risk of death by natural causes also showed particular elevation in the first 3 months. Considering gender, the relative increase in risk for external causes, including suicide, was higher for working-age women than for working-age men when compared with their general population counterparts. In the working-age group, discharged patients registered at practices in the least deprived localities had a higher risk of dying by suicide than those in the most deprived quintiles, although an association in the opposite direction in relation to practice-level deprivation was identified for death by natural causes in older adults.

### Comparison with existing evidence and interpretation

The elevated risk for each cause of death examined in the working-age population, particularly in the first three months after discharge, was similar to the results reported in a study conducted using national interlinked Danish registry data, although the relative risk for all-cause mortality was lower and that for suicide was higher in our study.^13^ This may reflect differences in the age profiles of the two respective study cohorts; the mean age at discharge in our working-age group was 15 years older than in the earlier Danish study cohort.^13^ Our findings reinforce previous recommendations for timely post-discharge support.^14,15^ Our study confirms previous findings that men have a higher absolute risk of dying by suicide.^3,5^ However, the findings that working-age women had the greatest elevation in suicide risk compared with matched general population comparators, a finding also noted in a recent Danish study,^16^ highlights the importance of maintaining a high level of support for both female and male patients post-discharge.

The lower absolute risk of suicide in discharged older adults in this population compared with working-age adults was not identified in previous meta-analyses.^2^ This may be partly explained by a survivor bias and a different diagnostic profile; over a third of the cohort had a primary diagnosis of dementia among whom fewer than five suicide deaths were reported. Although there is evidence that risk of suicide by people with dementia is elevated shortly after diagnosis and in people under 75,17 it is likely that many of those with a primary diagnosis of dementia in our study cohort, who have a median age of 80, are in later stages of dementia and may not possess the insight or capacity to plan a suicide.^18^ Although suicide is an important concern in this age group, with the highest relative risk of causes examined, it accounts for less than 3% of deaths, similar to the number of accidental deaths.

The overwhelming majority (94%) of deaths among older discharged patients were from natural causes. This is perhaps not unexpected considering the age profile and preponderance of dementia and may reflect underlying severity and physical comorbidity. Prioritising areas leading to the main causes of death – cardiovascular health and the management of dementia, in line with existing National Institute for Health and Care Excellence guidance (including consideration of alternatives to hospital admission)19 - may improve outcomes. The management of dementia in people requiring psychiatric care will continue to increase in importance over time as the population ages further. Particular focus should be given in areas of relative deprivation where risk of death was higher.

The elevated risk of death by natural causes in the first 3 months after discharge identified in older adults with and without dementia was also identified in American Veterans with dementia discharged from in-patient psychiatric care.^20^ This is of concern and we suggest that follow-up support for older adults should be holistic, including social support, physical health assessment and adjustments to living arrangements. This is particularly important for patients with longer lengths of stay, which are more common in older adults (almost 70% of our older cohort were in hospital for more than 30 days), who may have more difficulty adjusting after discharge.^21,22^ Previous research has identified that individuals also consider loneliness, emotional readiness for discharge and communication as important elements of safe discharge.^23^ Similar studies with older adults may enhance our understanding of their support needs during this risky transition.

Contrary to findings in the general population,^24^ our analysis shows a greater risk of dying by suicide and other external causes for working-age people discharged in the least deprived practice areas compared with the most deprived. However, our findings are consistent with a previous study in Denmark where higher income was associated with higher risk of suicide after discharge^25^ and a study that found lower deprivation to be associated with suicide after hospital admission for self-harm in England.^26^ It may be that people from affluent areas experience higher self-stigma as a result of the perceived loss of social status and differences to their peers than those in deprived areas.^27,28^ Deprivation was, however, measured at the area level rather than individual level, so it is possible that in less deprived but socioeconomically unequal areas people who are admitted to hospital have a lower socioeconomic position. It is also conceivable that health services in more deprived areas with higher incidence of severe mental ill health are better equipped to provide appropriate support.^25^ More research is needed to enhance the understanding of how people in this cohort access primary and emergency care, as this may indicate whether planned care is sufficient.

### Strengths and limitations

A strength of this large cohort study of routinely collected inter-linked healthcare and mortality records is that it has abundant statistical power for examining a range of rare mortality outcomes. The inclusion of both working-age and older adult patient groups as separate subcohorts enabled direct comparison of risk, with further stratification by gender and relative deprivation at the practice level. Linkage to primary care records allowed for direct comparison with people in the wider community. The broadly representative data-set allows for our findings to be generalised to all adults in England experiencing psychiatric hospital admissions.

This study has some limitations. Using routinely collected data limited the information that we could gather; for example, we had no measures of individual socioeconomic position or changes in social circumstances because of admission to hospital. In addition, although HES APC data should contain records of all NHS funded care in England, up to 15% of mental healthcare trusts did not contribute to the data-set in some years, meaning there may be some patients with recent admission history in the matched comparison group. However, any such misclassification would likely result in relative risk attenuation. Furthermore, because discharge information is only available in the CPRD linkage from 1997, it was not possible to ascertain whether the first discharge in the study period represented the true first discharge experienced by an individual. Therefore, the study is not directly comparable with investigations that considered only the first discharge in a person’s life. However, by including a ‘look-back’ period before the study start date, we ensured that the discharges were at least the first in 3 years.

### Implications for clinicians, policymakers and researchers

Although we have suggested possible explanations for our findings, this study is essentially descriptive, and our study design does not enable causal inference. Arguably, the experience of hospital admission itself may influence suicide risk post-discharge for some people.^29^ It is therefore important that psychiatric in-patient environments are as therapeutic as is possible and that future research considers the mechanisms by which a hospital stay may contribute to this risk. However, people who are admitted are acutely unwell and it is therefore not possible to know what their outcomes would have been had they not been admitted to hospital. Further research is needed to compare outcomes for people with severe mental illness who have been treated in the community, particularly as current policy emphasises alternatives to hospital admission.^30^ Our study has confirmed that the first months post-discharge are the riskiest in terms of suicide, re-emphasising the importance of timely follow-up. It has also identified that discharged older adults are at elevated risk of dying by natural causes in the early discharge period. In addition, our findings relating to high relative risks among recently discharged women, and those registered with practices in more affluent localities, show the importance of providing adequate support to all people discharged irrespective as to their perceived risk. Thus, therapeutic in-patient care and well-coordinated multiagency, person-centred discharge planning and follow-up support should be in place for all discharged patients. Older adults in particular should receive support to manage their physical health and activities of daily living, as well as their risk of dying by suicide.

## Supplementary Material

supplementary file

## Figures and Tables

**Table 1:** Sociodemographic and clinical characteristics of people discharged from in-patient mental healthcare at index discharge

	Working-age group, 18–64 years, *n* (%) (*n* = 67 559)	Older adults, ≥65 years, *n* (%) (*n* = 33 202)	Total, *n* (%) (*n* = 100 761)
Gender			
Male	36 854 (54.6)	12 999 (39.2)	49 853 (49.5)
Female	30 705 (45⋅4)	20 203 (60.8)	50 908 (50.5)
Age at discharge, years			
18–24	9212 (13.6)	–	9212 (9.1)
25–34	15 907 (23.5)	–	15 907 (15.8)
35–44	17 852 (26.4)	–	17 852 (17.7)
45–54	14 580 (21.6)	–	14 580 (14.5)
55–64	10 008 (14.8)	–	10 008 (9.9)
65–74	–	10 899 (32.8)	10 899 (10.8)
75–84	–	14 530 (43.8)	14 530 (14.4)
≥85	–	7 773 (23.4)	7 773 (7.7)
Ethnicity			
White	58 562 (86.7)	31 530 (95.0)	90 092 (89.4)
Black	3520 (5.2)	425 (1.3)	3945 (3.9)
Asian	2687 (4.0)	321 (1.0)	3008 (3.0)
Mixed	922 (1.4)	62 (0.2)	984 (1.0)
Other	1121 (1.7)	153 (0.5)	1274 (1.3)
Unknown	747 (1.1)	711 (2.1)	1458 (1.4)
Index of Multiple Deprivation quintile: practice level			
1 (least deprived)	8041 (11–9)	5211 (15.7)	13 252 (13.2)
2	9632 (14.3)	5493 (16.5)	15 125 (15.0)
3	11 980 (17.7)	6605 (19.9)	18 585 (18.4)
4	17 376 (25.7)	7797 (23.5)	25 173 (25.0)
5 (most deprived)	20 530 (30.4)	8096 (24–4)	28 626 (28.4)
Prior self-harm in previous 6 months	11 518 (17.0)	2145 (6.5)	13 663 (13.6)
Number of comorbidities at discharge			
0	58 220 (86.2)	14 208 (42.8)	72 428 (71.9)
1	7058 (10.4)	10 022 (30.2)	17 080 (17.0)
2	1704 (2.5)	5170 (15.6)	6874 (6.8)
3 or more	577 (0.9)	3802 (11.5)	4379 (4.3)
Primary diagnosis at discharge			
Dementia	508 (0.8)	11 549 (34.8)	12 057 (12.0)
Other organic disorders	584 (0.9)	833 (2.5)	1417 (1.4)
Psychoactive substance misuse disorder	12 203 (18.1)	567 (1.7)	12 770 (12.7)
Schizophrenia and related disorders	11 968 (17.7)	2440 (7.3)	14 408 (14.3)
Bipolar disorder	4480 (6.6)	1391 (4.2)	5871 (5.8)
All other mood (affective) disorders	12 351 (18.3)	68 66 (20.7)	19 217 (19.1)
Anxiety disorders	7514 (11.1)	1665 (5.0)	9179 (9.1)
Eating disorder	566 (0.8)	7 (0.0)	573 (0.6)
Personality disorder	2650 (3.9)	119 (0.4)	2769 (2.7)
Other mental health diagnosis	1748 (2.6)	551 (1.7)	2299 (2.3)
Unspecified/all other	12 987 (19.2)	7214 (21.7)	20 201 (20.0)
Length of index hospital admission			
Under 7 days	20 182 (29.9)	2295 (6.9)	22 477 (22.3)
1 week to 30 days	27 208 (40.3)	8060 (24.3)	35 268 (35.0)
30 days to 6 months	18 228 (27.0)	20 933 (63.0)	39 161 (38.9)
over 6 months	1921 (2.8)	1908 (5.7)	3829 (3.8)
Missing data	20 (0.0)	6 (0.0)	26 (0.0)
Destination type			
Usual place of residence	63 177 (93.5)	23 083 (69.5)	86 260 (85.6)
Temporary residence	3515 (5.2)	998 (3.0)	4513 (4.5)
Care home/Other residential	867 (1.3)	9121 (27.5)	9988 (9.9)
Discharge method			
Discharged with clinical consent	63 020 (93.3)	32 260 (97.2)	95 280 (94.6)
Self/relative discharge	3657 (5.4)	458 (1.4)	4115 (4.1)
Discharged by Mental Health review tribunal	214 (0.3)	80 (0.2)	294 (0.3)
Missing data	668 (1.0)	404 (1.2)	1072 (1.1)

**Table 2:** Cumulative incidence by cause of death in the discharged and matched comparison cohorts at 1-year post-discharge from in-patient care, by age group and gender

	Working-age adults (18–64)									Older adults (65+)							
	All			Female			Male			All			Female			Male	
	*n*	Cumulative incidence, % (95% CI)		*n*	Cumulative incidence, % (95% CI)		*n*	Cumulative incidence, % (95% CI)		*n*	Cumulative incidence, % (95% CI)		*n*	Cumulative incidence, % (95% CI)		*n*	Cumulative incidence, % (95% CI)
Discharged cohort	67 559			30 705			36 854			33 202			20 203			12 999	
All causes	1301	2.1 (2.0–2.3)		440	1.6 (1.4–1.7)		861	2.6 (2.5–2.8)		3473	14.1 (13.6–14.5)		1804	11.9 (11.4–12.4)		1669	17.5 (16.8–18.3)
Natural causes	628	1.0 (1.0–1.1)		228	0.8 (0.7–0.9)		400	1.2 (1.1–1.4)		3281	13.3 (12.9–13.7)		1711	11.3 (10.8–11.8)		1570	16.5 (15.8–17.3)
External causes	673	1.1 (1.0–1.2)		212	0.8 (0.7–0.9)		461	1.4 (1.3–1.5)		192	0.8 (0.7–0.9)		93	0.6 (0.5–0.7)		99	1.0 (0.8–1.2)
Suicide	517	0.8 (0.8–0.9)		158	0.6 (0.5–0.6)		359	1.1 (1.0–1.2)		98	0.4 (0.3–0.5)		38	0.2 (0.2–0.3)		60	0.6 (0.5–0.8)
Accidental death	155	0.3 (0.2–0.3)		54	0.2 (0.1–0.3)		101	0.3 (0.3–0.4)		93	0.4 (0.3–0.5)		55	0.4 (0.3–0.5)		38	0.4 (0.3–0.5)
Alcohol-specific death	140	0.2 (0.2–0.3)		57	0.2 (0.2–0.3)		83	0.3 (0.2–0.3)		–	–		–	–		–	–
Drug-related death	188	0.3 (0.3–0.4)		63	0.2 (0.2–0.3)		125	0.4 (0.3–0.4)		–	–		–	–		–	–
Comparison cohort	1 331 387			603 946			727 441			568 534			355 506			213 028	
All causes	2560	0.2 (0.2–0.2)		985	0.2 (0.2–0.2)		1575	0.2 (0.2–0.2)		26 838	4.8 (4.7–4.9)		16 193	4.6 (4.6–4.7)		10 645	5.1 (5.0–5.2)
Natural causes	2288	0.2 (0.2–0.2)		927	0.2 (0.2–0.2)		1361	0.2 (0.2–0.2)		26 317	4.7 (4.7–4.8)		15 881	4.5 (4.5–4.6)		10 436	5.0 (4.9–5.1)
External causes	272	0.02 (0.02–0.02)		58	0.01 (0.01–0.01)		214	0.03 (0.03–0.03)		521	0.09 (0.09–0.1)		312	0.09 (0.08–0.1)		209	0.1 (0.09–0.1)
Suicide	126	0.01 (0.01–0.01)		21	0.004 (0.002–0.01)		105	0.02 (0.01–0.02)		39	0.01 (0.01–0.01)		14	0.004 (0.002–0.01)		25	0.01 (0.01–0.02)
Accidental death	135	0.01 (0.01–0.01)		34	0.01 (0.004–0.01)		101	0.01 (0.01–0.02)		452	0.08 (0.07–0.09)		282	0.08 (0.07–0.09)		170	0.08 (0.07–0.09)
Alcohol-specific death	126	0.01 (0.01–0.01)		36	0.01 (0.004–0.01)		90	0.01 (0.01–0.02)		–	–		–	–		–	–
Drug-related death	78	0.01 (0.005–0.01)		21	0.004 (0.002–0.01)		57	0.01 (0.01–0.01)		–	–		–	–		–	–

**Table 3:** Incidence rates and relative risks for external and natural causes of death by practice-level Index of Multiple Deprivation (IMD) quintile and age group during the first year after discharge

			External causes of death				Natural causes of death		
	Number of people discharged	Person-years	Deaths	Incidence rate, per 100 000	Hazard ratio^a^ (95% CI)		Deaths	Incidence rate, per 100 000	Hazard ratio^a^ (95% CI)
Working age									
Least deprived, 1	8041	7058	110	1559	1		83	1176	1
2	9632	8432	111	1316	0.85 (0.65–1.10)		84	996	0.86 (0.64–1.17)
3	11 980	10 554	119	1127	0.71 (0.55–0.93)		122	1156	1.01 (0.76–1.34)
4	17 376	15 262	153	1003	0.64 (0.50–0.81)		173	1134	1.03 (0.79–1.33)
Most deprived, 5	20 530	18 069	180	996	0.63 (0.50–0.80)		166	919	0.86 (0.66–1.12)
Older adults									
Least deprived, 1	5211	3378	24	711	1		450	13 323	1
2	5493	3606	29	804	1.12 (0.65–1.93)		502	13 923	1.07 (0.94–1.21)
3	6605	4390	46	1048	1.48 (0.91–2.43)		671	15 284	1.16 (1.03–1.30)
4	7797	5390	42	779	1.10 (0.66–1.81)		790	14 656	1.13 (1.01–1.27)
Most deprived, 5	8096	5593	51	912	1.27 (0.78–2.07)		868	15 520	1.24 (1.10–1.38)

a Hazard ratio (HR) baseline is IMD1 (least deprived); HRs adjusted for gender and age (natural causes modelled using age as a linear variable, external causes in working-age modelled using a non-linear age variable and external causes in older age modelled using age by broad age group (65–74 and ≥75 years) based on the best-fitting model).

## Data Availability

The clinical codes used in this study are available online at https://clinicalcodes.rss.mhs.man.ac.uk/. The codes are also available from the corresponding author on request. Access to data is available only once approval has been obtained through the individual constituent entities controlling access to the data. The primary care data can be requested via application to the Clinical Practice Research Datalink.
