# Supplementary material for: Suicide and other causes of death among working-age and older adults in the year after discharge from in-patient mental healthcare in England: matched cohort study
Source: Br J Psychiatry. Author manuscript; Available in PMC 2023 Feb 1. (PMC7613106; doi:10.1192/bjp.2021.176)
Supplement: supplementary file [file EMS138180-supplement-supplementary_file.docx]

**Supplementary Appendix**

**Contents**

**Page 1** Datasets used with key links and references

**Page 2 Table S1** Delineation of the cohort of recently discharged patients

**Page 3** Sensitivity Analyses

**Page 4 Table S2** RECORD Statement for observational studies

**Page 8 Table S3** Cumulative incidence by cause of death in discharged and matched

comparison older adult cohorts at 1-year post-discharge from inpatient care,

by age group and gender - excluding Primary diagnosis of Dementia

**Page 9 Figure S1** Relative risk estimates comparing discharged older adults with their general

population counterparts; breakdown by dementia diagnosis.

**Page 10 Table S3** Hazard ratios between discharged working age and older adults and their

respective comparison cohorts, with testing for effect modification by gender, at one year after discharge

**Page 11** **Figure S1** Relative risk of suicide in people discharged from psychiatric inpatient care

by age group and gender during the first year after discharge

**Page 11 Figure S2** Risk of suicide in working age people discharged from psychiatric inpatient

care between practice-level IMD quintiles during the first year after discharge

**Page 12 Figure S3** Risk of death by natural causes in older adults discharged from psychiatric

inpatient care between practice-level IMD quintiles during the first year after discharge

**S1. Datasets used with key links and references**

**CPRD primary care dataset (GOLD)**

Herrett E, Gallagher AM, Bhaskaran K, et al. Data Resource Profile: Clinical Practice Research Datalink (CPRD). *Int J Epidemiol* 2015; **44**(3): 827-36. <https://academic.oup.com/ije/article/44/3/827/632531>

**CPRD primary care dataset (Aurum)**

Wolf A, Dedman D, Campbell J, et al. Data resource profile: Clinical Practice Research Datalink (CPRD) Aurum. *International Journal of Epidemiology* 2019; **dyz034**. <https://academic.oup.com/ije/article/48/6/1740/5374844>

**HES Secondary Care dataset**

The Admitted Patient Care or Inpatient dataset is part of a collection of datasets in the Hospital Episode Statistics (HES) data warehouse containing information on all admissions to English NHS hospitals. It is derived from the Commissioning Dataset (CDS) which is used as the basis of allocating payment for care provided by hospitals. It is administered by NHS Digital who facilitate the data-linkage to the CPRD.

NHS Digital. Hospital Episode Statistics - Hospital Admitted Patient Care Activity [Datafiles]. 2009-2019. <https://digital.nhs.uk/data-and-information/publications/statistical/hospital-admitted-patient-care-activity> (accessed Nov 14 2019).

Further information about the linkage process and quality checks can be found in the article below.

Padmanabhan S, Carty L, Cameron E, Ghosh RE, Williams R, Strongman H. Approach to record linkage of primary care data from Clinical Practice Research Datalink to other health-related patient data: overview and implications. *European Journal of Epidemiology* 2019; **34**(1): 91-9.

**Index of Multiple Deprivation (IMD)**

The IMD is a composite measure of relative deprivation, encompassing seven domains: health, income, employment, education, crime, barriers to housing and services, and living environment.

Ministry of Housing, Communities & Local Government,. English indices of deprivation [Intenet]. 26/09/2019 2019. <https://www.gov.uk/government/collections/english-indices-of-deprivation> (accessed Nov 25 2019).

**Office for National Statistics – Mortality Records**

<https://www.ons.gov.uk/peoplepopulationandcommunity/birthsdeathsandmarriages/deaths>

**Alcohol related deaths**

Office for National Statistics. Alcohol-specific deaths in the UK: registered in 2017. 2018. <https://www.ons.gov.uk/peoplepopulationandcommunity/healthandsocialcare/causesofdeath/bulletins/alcoholrelateddeathsintheunitedkingdom/registeredin2017> (accessed Nov 10 2019).

**Drug Specific deaths**

Office for National Statistics. Deaths related to drug poisoning in England and Wales: 2018 registrations. 2019. <https://www.ons.gov.uk/peoplepopulationandcommunity/birthsdeathsandmarriages/deaths/bulletins/deathsrelatedtodrugpoisoninginenglandandwales/2018registrations> (accessed Oct 09 2019).

**Dementia Diagnosis (ICD-10)**

Codes for dementia classification in HES were taken from the following study which also used the CPRD and HES datasets:

Muzambi R, Bhaskaran K, Smeeth L, Brayne C, Chaturvedi N, Warren-Gash C. Assessment of common infections and incident dementia using UK primary and secondary care data: a historical cohort study. *The Lancet Healthy Longevity* 2021; **2**(7): e426-e35.

**Other codelists**

All other codelists can be found at <https://clinicalcodes.rss.mhs.man.ac.uk/>

**Table S1- Delineation of the cohort of recently discharged patients**

All numbers tabulated are those patients who remain in the cohort after the exclusion criteria have been applied. Patients can have multiple episodes of care within one hospital stay, and/or several episodes across more than one hospital stay. The original data provided by the CPRD contained all episodes for each patient to identify the index episode. Aurum contains records from practices using the EMIS Web® patient record system and GOLD contains records from practices using Vision®.

|  |  | **Aurum Dataset** | | **GOLD Dataset** | |
| --- | --- | --- | --- | --- | --- |
| **Criteria** | **Further detail** | **Patients** | **Patient Episodes** | **Patients** | **Patient Episodes** |
| **HES Cohort delineation** |  |  |  |  |  |
| Records received from CPRD. All episodes of care under a psychiatric consultant between 01/01/1998 and 31/05/2019 | Main Specialty= 710/711/712/713/715 | 485,715 | 1,588,855 | 193,317 | 623,756 |
| **Numbers after exclusions** | | | | | |
| Removal of patients from duplicate practices which have moved electronic record systems (GOLD only) |  | 485,715 | 1,588,855 | 123,461 | 402,426 |
| Keep only ‘ordinary’ inpatients  (Exclude day patients) | (classification!=1) | 484,690 | 1,578,462 | 123,211 | 398,609 |
| Limit to episodes that end with a community discharge | Keep  Discharge destination = 19 Normal place of residence  Or 29 = Temporary place of residence  Or 54,65,66,85 = care homes/foster care  Exclude all transfers and any episodes where the discharge method is 8 (N/A still in hospital) or 4 (died in hospital) | 456,355 | 1,184,355 | 115,812 | 301,716 |
| Limit to discharges within the study period, without recent prior hospitalisation. | Exclude all patients whose  -only discharge falls outside the study period (before 1^st^ January 2001, after 31^st^ May 2018)  -who have a first discharge before the study period and a second within three years of the beginning of the study period. | 365,058 | 799,615 | 92,042 | 202,096 |
| Limit to first discharge | Excludes all subsequent episodes of care to leave one episode per patient | 365,058 | 365,058 | 91,108 | 91,108 |
| Exclude patients with poor quality HES data completion | -episode end and discharge end do not match & either the discharge method or discharge destination is unknown  -exclude those where both the discharge destination and discharge method are unknown.  -drop patients where the episode end date is before the discharge date, or it is missing. | 360,583 |  | 90,979 |  |
| **Linkage Availability** | | | |  |  |
| Exclude patients not eligible for linkage to all necessary data files | -not present in CPRD denominator file  -no linkage with HES,ONS, IMD) | 356,606 |  | 90,150 |  |
| **CPRD Primary Care dataset exclusions** | | | |  |  |
| Exclude patients without acceptable records. | Acceptable!=1 (Aurum)  Accept!=1 (GOLD)  Excludes any with key data missing such as year of birth and gender | 323,526 |  | 84,352 |  |
| Cohort inclusion criteria. | Exclude if:   - Not registered at the GP practice, or had less than 6 months registered at the practice at point of discharge - Age under 10 - Patient recorded at more than one practice at time of discharge (duplicates) | 87,608 |  | 19,795 |  |
| **Further Exclusions after matching and full linkage to all datasets** | | | |  |  |
| Drop if ONS date of death is before the index date. |  | 87,604 |  | 19,795 |  |
| Drop if case has no matches |  | 87,442 |  | 19,749 |  |
| Drop if case does not meet final quality criteria | -Episode end after discharge date  -Discharge destination is unknown | 84,102 |  | 19,095 |  |
| Excluding those under 18 |  | 82,103 |  | 18,658 |  |
| Final Combined Cohort |  | 100,761 | | | |

**S2 - Sensitivity Analyses**

Four sensitivity analyses were carried out:

1. Apply interval censoring for periods of readmission to inpatient psychiatric care to assess any difference in relative suicide risk estimates, as admission may be protective against suicide.

In order to account for readmission, the exposure variable ‘discharge from psychiatric inpatient care’ was made to be time varying. Periods after discharge when an individual was in the community were set as being at risk. Periods when an individual was back in hospital were set as not being at risk at all – in order to set the most extreme parameters for the sensitivity analysis. Individuals were in effect interval censored - an individual left the study when they were readmitted to inpatient psychiatric care and re-entered on subsequent discharge. The binary exposed/unexposed variable was set to missing for all lines where an individual was censored due to readmission. After data was split, the data was set for analysis using the Stset command as for the primary analysis and cox regression run to calculate hazard ratios. Any line where the exposure variable was set to missing was automatically excluded in the regression calculation.

Models excluding periods of readmission to psychiatric inpatient care showed very small, non-significant, increases in relative risk of suicide, from a HR of 89 to 91 in working adults and from 59 to 61 in older adults.

1. Exclude patients in the older adult cohort with a primary diagnosis of dementia to assess any difference in absolute and relative risk estimates

All absolute and relative risks of all-cause, external or natural cause mortality and suicide were calculated for older adults excluding those with a primary diagnosis of dementia. This had some impact on overall risk estimates, reducing the absolute and relative risk of death by natural causes and increasing slightly the risk of external causes (see Table S3 and Figure S1). However, the findings compared to working age adults and timing of death after discharge were unchanged.

3) Exclude the final year of discharges from analyses to estimate the impact of delayed death registrations in that latest year of observation;^1^

Sensitivity analysis, excluding the final year of discharges in the observation period, showed little change in estimates. The risk of suicide in working-age adults increased by 0.01% and decreased by the same amount in older adults.

4) Exclude individuals with no documented overnight stay.

Although regular day patients were excluded from the primary analysis (analysis was limited to those classified as regular inpatients) a total of 1,902 individuals who had an episode of care under a psychiatric consultant were included who had ‘zero’ days of care. This could be valid: for example due to self-discharge or individuals who were admitted for observation and then discharged. However in some cases it could be a misclassification of a patient as an inpatient. Sensitivity analysis excluding these individuals was carried out. For the one year period post-discharge the cumulative incidence and hazard ratios were identical or changed only very slightly. Therefore these individuals were kept in the main analysis.

**Table S3 - The RECORD statement^2^ – checklist of items, extended from the STROBE statement that should be reported in observational studies using routinely collected health data.**

|  | **Item No.** | **STROBE items** | **Location in manuscript where items are reported** | **RECORD items** | **Location in manuscript where items are reported** |
| --- | --- | --- | --- | --- | --- |
| **Title and abstract** | | | | | |
|  | 1 | (a) Indicate the study’s design with a commonly used term in the title or the abstract (b) Provide in the abstract an informative and balanced summary of what was done and what was found |  | RECORD 1.1: The type of data used should be specified in the title or abstract. When possible, the name of the databases used should be included.  RECORD 1.2: If applicable, the geographic region and timeframe within which the study took place should be reported in the title or abstract.  RECORD 1.3: If linkage between databases was conducted for the study, this should be clearly stated in the title or abstract. | Abstract  Geographic region – in title  Timeframe – in abstract  Abstract |
| **Introduction** | | | | | |
| Background rationale | 2 | Explain the scientific background and rationale for the investigation being reported |  |  | Introduction: paragraphs 1-2, |
| Objectives | 3 | State specific objectives, including any prespecified hypotheses |  |  | Introduction: paragraph 3-4 |
| **Methods** | | | | | |
| Study Design | 4 | Present key elements of study design early in the paper |  |  | Methods: ‘Study population and design’ |
| Setting | 5 | Describe the setting, locations, and relevant dates, including periods of recruitment, exposure, follow-up, and data collection |  |  | Methods: ‘Data source’ and ‘Study population and design’ |
| Participants | 6 | *(a) Cohort study* - Give the eligibility criteria, and the sources and methods of selection of participants. Describe methods of follow-up  *Case-control study* - Give the eligibility criteria, and the sources and methods of case ascertainment and control selection. Give the rationale for the choice of cases and controls  *Cross-sectional study* - Give the eligibility criteria, and the sources and methods of selection of participants  *(b) Cohort study* - For matched studies, give matching criteria and number of exposed and unexposed  *Case-control study* - For matched studies, give matching criteria and the number of controls per case |  | RECORD 6.1: The methods of study population selection (such as codes or algorithms used to identify subjects) should be listed in detail. If this is not possible, an explanation should be provided.  RECORD 6.2: Any validation studies of the codes or algorithms used to select the population should be referenced. If validation was conducted for this study and not published elsewhere, detailed methods and results should be provided.  RECORD 6.3: If the study involved linkage of databases, consider use of a flow diagram or other graphical display to demonstrate the data linkage process, including the number of individuals with linked data at each stage. | Methods: ‘Study population and design’  Methods: ‘Study population and design’ ‘Appendix pg2’ |
| Variables | 7 | Clearly define all outcomes, exposures, predictors, potential confounders, and effect modifiers. Give diagnostic criteria, if applicable. |  | RECORD 7.1: A complete list of codes and algorithms used to classify exposures, outcomes, confounders, and effect modifiers should be provided. If these cannot be reported, an explanation should be provided. | Methods: ‘Classification of outcomes and covariates’ |
| Data sources/ measurement | 8 | For each variable of interest, give sources of data and details of methods of assessment (measurement).  Describe comparability of assessment methods if there is more than one group |  |  | Methods: ‘data sources’ and ‘Classification of outcomes and covariates’ |
| Bias | 9 | Describe any efforts to address potential sources of bias |  |  | Methods: ‘data analysis’ |
| Study size | 10 | Explain how the study size was arrived at |  |  | Methods: ‘study population’*.*  *Appendix Table S1* |
| Quantitative variables | 11 | Explain how quantitative variables were handled in the analyses. If applicable, describe which groupings were chosen, and why |  |  | Methods: data analysis |
| Statistical methods | 12 | (a) Describe all statistical methods, including those used to control for confounding  (b) Describe any methods used to examine subgroups and interactions  (c) Explain how missing data were addressed  (d) *Cohort study* - If applicable, explain how loss to follow-up was addressed  *Case-control study* - If applicable, explain how matching of cases and controls was addressed  *Cross-sectional study* - If applicable, describe analytical methods taking account of sampling strategy  (e) Describe any sensitivity analyses |  |  | Methods: Statistical analyses (a,b)  Classification of outcomes and covariates (c)  Study population and design(c,d)  Supplementary material S2 (e) |
| Data access and cleaning methods |  | .. |  | RECORD 12.1: Authors should describe the extent to which the investigators had access to the database population used to create the study population.  RECORD 12.2: Authors should provide information on the data cleaning methods used in the study. | Methods: ‘Study population and design’ and ‘Role of funding source’  Appendix Table S1 |
| Linkage |  | .. |  | RECORD 12.3: State whether the study included person-level, institutional-level, or other data linkage across two or more databases. The methods of linkage and methods of linkage quality evaluation should be provided. | Methods: ‘Data source’  And ‘Study population and design’ |
| **Results** | | | | | |
| Participants | 13 | (a) Report the numbers of individuals at each stage of the study (*e.g.*, numbers potentially eligible, examined for eligibility, confirmed eligible, included in the study, completing follow-up, and analysed)  (b) Give reasons for non-participation at each stage.  (c) Consider use of a flow diagram |  | RECORD 13.1: Describe in detail the selection of the persons included in the study (*i.e.,* study population selection) including filtering based on data quality, data availability and linkage. The selection of included persons can be described in the text and/or by means of the study flow diagram. | Methods; ‘Study population and design’, appendix S1 and results paragraph 1. |
| Descriptive data | 14 | (a) Give characteristics of study participants (*e.g.*, demographic, clinical, social) and information on exposures and potential confounders  (b) Indicate the number of participants with missing data for each variable of interest  (c) *Cohort study* - summarise follow-up time (*e.g.*, average and total amount) |  |  | Results, table 1, table 3 |
| Outcome data | 15 | *Cohort study* - Report numbers of outcome events or summary measures over time  *Case-control study* - Report numbers in each exposure category, or summary measures of exposure  *Cross-sectional study* - Report numbers of outcome events or summary measures |  |  | Results, paragraph 2 and Table 2. |
| Main results | 16 | (a) Give unadjusted estimates and, if applicable, confounder-adjusted estimates and their precision (e.g., 95% confidence interval). Make clear which confounders were adjusted for and why they were included  (b) Report category boundaries when continuous variables were categorized  (c) If relevant, consider translating estimates of relative risk into absolute risk for a meaningful time period |  |  | Results, paragraph 2-4, table 2, figure 1 |
| Other analyses | 17 | Report other analyses done—e.g., analyses of subgroups and interactions, and sensitivity analyses |  |  | Results, paragraph 5-6. Table 3, figure 2&3. Appendix Table S4 |
| **Discussion** | | | | | |
| Key results | 18 | Summarise key results with reference to study objectives |  |  | Discussion, paragraph 1 |
| Limitations | 19 | Discuss limitations of the study, taking into account sources of potential bias or imprecision. Discuss both direction and magnitude of any potential bias |  | RECORD 19.1: Discuss the implications of using data that were not created or collected to answer the specific research question(s). Include discussion of misclassification bias, unmeasured confounding, missing data, and changing eligibility over time, as they pertain to the study being reported. | Discussion, paragraph 7 |
| Interpretation | 20 | Give a cautious overall interpretation of results considering objectives, limitations, multiplicity of analyses, results from similar studies, and other relevant evidence |  |  | Discussion: paragraphs 2-5 |
| Generalisability | 21 | Discuss the generalisability (external validity) of the study results |  |  | Discussion paragraph 6 |
| **Other Information** | | | | | |
| Funding | 22 | Give the source of funding and the role of the funders for the present study and, if applicable, for the original study on which the present article is based |  |  | Methods, ‘Role of funding source’, ‘Acknowledgements’ |
| Accessibility of protocol, raw data, and programming code |  | .. |  | RECORD 22.1: Authors should provide information on how to access any supplemental information such as the study protocol, raw data, or programming code. | Data sharing, |

*Checklist is protected under Creative Commons Attribution ([CC BY](http://creativecommons.org/licenses/by/4.0/)) license.

Reference: Benchimol EI, Smeeth L, Guttmann A, et al. The REporting of studies Conducted using Observational Routinely-collected health Data (RECORD) Statement. *PLOS Medicine* 2015; **12**(10): e1001885.

**Table S3 - Cumulative incidence by cause of death in discharged and matched comparison older adult cohorts at 1-year post-discharge from inpatient care, by age group and gender - excluding Primary diagnosis of Dementia**

|  | Total |  |  | Female |  |  | Male |  |  |
| --- | --- | --- | --- | --- | --- | --- | --- | --- | --- |
|  | n | Cumulative incidence, per 100,000 (95%CI) | | n | Cumulative incidence, per 100,000 (95%CI) | | n | Cumulative incidence, per 100,000 (95%CI) | |
| **Discharge Cohort** | **N=21,653** | | | **N=13,359** | |  | **N=8,294** | | |
| All-cause Mortality | **2,010** | 11.1% | (10.6-11.5) | **1,032** | 9.2% | (8.6-9.7) | **978** | 14.2% | (13.4-15) |
| Natural Causes | **1,885** | 10.2% | (9.8-10.7) | **961** | 8.5% | (8-9.1) | **894** | 13.0% | (12.2-13.8) |
| External | **155** | 0.8% | (0.7-1) | **71** | 0.6% | (0.5-0.8) | **84** | 1.2% | (1-1.5) |
| Suicide | **95** | 0.5% | (0.4-0.6) | **37** | 0.3% | (0.2-0.4) | **58** | 0.8% | (0.6-1.1) |
| Accidents | **59** | 0.3% | (0.3-0.4) | **34** | 0.3% | (0.2-0.4) | **25** | 0.4% | (0.2-0.5) |
| **Matched Comparison Cohort** | **N=379,994** | |  | **N=239,263** | |  | **N=140,731** | | |
| All-cause Mortality | **15,498** | 4.1% | (4.1-4.2) | **9,296** | 4.0% | (3.9-4) | **6,202** | 4.5% | (4.4-4.6) |
| Natural Causes | **15,226** | 4.1% | (4-4.1) | 9,132 | 3.9% | (3.8-4) | **6,094** | 4.4% | (4.3-4.5) |
| External | **272** | 0.1% | (0.1-0.1) | **164** | 0.1% | (0.1-0.1) | **108** | 0.1% | (0.1-0.1) |
| Suicide | **26** | 0.01% | (0.01-0.01) | **13** | 0.01% | (0.003-0.01) | **13** | 0.0% | (0.01-0.02) |
| Accidents | **235** | 0.1% | (0.1-0.1) | **146** | 0.1% | (0.1-0.1) | **89** | 0.1% | (0.05-0.08) |

**Figure S1 - Relative risk estimates comparing discharged older adults with their general population counterparts; breakdown by dementia diagnosis**

**Table S4** **Hazard ratios between discharged working age and older adults and their respective comparison cohorts, with testing for effect modification by gender, at one year after discharge**

|  | **Working-age men** | | **Working-age women** | | **Total** | |  |  |  |
| --- | --- | --- | --- | --- | --- | --- | --- | --- | --- |
|  | **Hazard Ratio*** | **(95% CI)** | **Hazard Ratio*** | **(95% CI)** | **Hazard Ratio*** | **(95% CI)** | **Interaction term  HR (women compared to men)** | **(95% CI)** |  |
| **All causes** | 11.3 | (10.4-12.3) | 8.9 | (7.9-10.0) | 10.4 | (9.7-11.1) | 0.8 | (0.7-0.9) | p=0.001 |
| **Natural causes** | 6.0 | (5.4-6.7) | 4.8 | (4.1-5.5) | 5.5 | (5.0-6.0) | 0.8 | (0.7-1.0) | p=0.014 |
| **External causes** | 45.7 | (38.5-54.2) | 89.3 | (64.1-124.5) | 53.8 | (46.3-62.5) | 1.8 | (1.3-2.6) | p=0.001 |
| **Suicide** | 74.5 | (59.0-94.2) | 163.9 | (100.3-267.7) | 89.5 | (72.6-110.2) | 2.2 | (1.3-3.8) | p=0.003 |
| **Accidental death** | 21.0 | (15.7-28.1) | 41.3 | (24.9-68.5) | 25.1 | (19.6-32.1) | 1.7 | (1.0-3.0) | p=0.044 |
| **Alcohol-specific death** | 20.3 | (14.7-28.0) | 31.1 | (20.1-48.1) | 23.5 | (18.2-30.4) | 1.5 | (0.9-2.6) | p=0.13 |
| **Drug-related death** | 46.8 | (33.3-65.7) | 89.2 | (48.0-165.8) | 55.6 | (41.6-74.4) | 1.7 | (0.9-3.3) | p=0.11 |
|  |  |  |  |  |  |  |  |  |  |
|  | **Older men (65 and over)** | | **Older women (65 and over)** | | **Total** | |  |  |  |
|  | Hazard Ratio* | **(95% CI)** | **Hazard Ratio*** | **(95% CI)** | **Hazard Ratio*** | **(95% CI)** | **Interaction term  HR (women compared to men)** | **(95% CI)** |  |
| **All causes** | 3.5 | (3.3-3.6) | 2.6 | (2.5-2.8) | 3.0 | (2.9-3.1) | 0.8 | (0.7-0.8) | P<0.001 |
| **Natural causes** | 3.3 | (3.1-3.5) | 2.6 | (2.4-2.7) | 2.9 | (2.7-3.0) | 0.8 | (0.7-0.8) | P<0.001 |
| **External causes** | 11.4 | (8.7-15.0) | 7.7 | (6.0-9.9) | 9.2 | (7.6-11.0) | 0.7 | (0.5-1.0) | p=0.036 |
| **Suicide** | 67.7 | (37.2-123.1) | 62.9 | (29.8-132.7) | 58.7 | (38.1-90.4) | 0.8 | (0.4-2.0) | p=0.70 |
| **Accidental death** | 4.9 | (3.3-7.2) | 5.1 | (3.7-7.0) | 5.0 | (3.9-6.4) | 1.1 | (0.6-1.7) | p=0.83 |

*Adjusted for patient-level IMD quintile

**Figure S2 – Relative risk of suicide in people discharged from psychiatric inpatient care by age group and gender during the first year after discharge**

* All analysis adjusted for Index of multiple deprivation (IMD) at patient level. Error bars show 95% Cis.

**Figure S3 - Risk of suicide in working age people discharged from psychiatric inpatient care between practice-level IMD quintiles during the first year after discharge**

*Adjusted for age (modelled as non-linear) and gender. IMD = Index of Multiple Deprivation. Error bars show 95% Cis. Baseline is least deprived

**Figure S4 - Risk of death by natural causes in older adults discharged from psychiatric inpatient care between practice-level IMD quintiles during the first year after discharge**

*Adjusted for age (modelled as non-linear) and gender. IMD = Index of Multiple Deprivation. Error bars show 95% Cis. Baseline is least deprived

**Supplementary Material: References**

1. Office for National Statistics. Impact of registration delays on mortality statistics: 2016 [Internet]. 2018. <https://www.ons.gov.uk/peoplepopulationandcommunity/birthsdeathsandmarriages/deaths/methodologies/impactofregistrationdelaysonmortalitystatistics2016> (accessed Sept 13 2019).

2. Benchimol EI, Smeeth L, Guttmann A et al. The REporting of studies Conducted using Observational Routinely-collected health Data (RECORD) Statement. *PLoS Medicine* 2015; in press.
